# Parental Control and College Students’ Adversarial Growth: A Discussion on Chinese One-Child Families

**DOI:** 10.3390/healthcare10101872

**Published:** 2022-09-26

**Authors:** Ting Nie, Gaoxi Hu, Tengfeng Qiu

**Affiliations:** 1 Management Department, School of Business, Macao University of Science and Technology, Macao 999078, China; 2 Department of Human Resource Management, College of Management, Zhongkai University of Agriculture and Engineering, Guangzhou 510225, China; 3 Department of Employment Relations, School of Public Administration, Guangdong University of Finance, Guangzhou 510520, China

**Keywords:** parental control, self-identity, adversarial growth, growth mindset, college student

## Abstract

Parental control can affect a children’s attitudes and their ability to cope with adversity after they become adults. This study explored the influence mechanism of parental control on adversity growth and the moderating effect of a growth mindset through a questionnaire survey completed by 354 Chinese college students born in one-child families. Hierarchical regression and structural equation analysis results show that parental control negatively affects adversarial growth, and self-identity plays a mediating role between parental control and adversarial growth. A higher degree of parental control will reduce the individual’s self-identity, which is not conducive to the occurrence of adversarial growth. A growth mindset negatively moderates the indirect effect of parental control on adversarial growth through self-identity. Individuals with a strong growth mindset have reduced negative effects of parental control on self-identity and adversarial growth. Even in countries with collectivist cultures, parental controls also need to be implemented carefully, and controlling parenting styles may be detrimental to individual growth after adversity. At the same time, it is necessary to consciously cultivate children’s growth mindsets, so as to inhibit the negative impact of parental control on adversarial growth.

## 1. Introduction

Parenting styles have a significant impact on children’s personality and behaviors in adulthood [1]. Especially when children are faced with adversity, there are significant differences in individuals’ immediate reactions and coping styles. Multiple studies have verified that positive parenting styles such as authoritative and empowering parenting can help children cope with adversity calmly, maintain a good mental state and psychological level, and gain opportunities for growth from adversity [2,3]. However, the role of controlling parenting has always been controversial. Parental control mainly emphasizes that parents force children to accept the parents’ own thoughts and emotions in order to limit children’s psychological and behavioral autonomy [4]. Parental control may be manifested in many aspects, and the most widely recognized in current research are psychological control and behavioral control [5]. Some studies have pointed out that parental behavior control is beneficial to children’s socialization and adaptive development, and provides necessary guidance for children [6]. However, most studies have confirmed that parental control hinders children’s personality development. In particular, parental psychological control can impair adolescent autonomy and self-control and is associated with externalizing problem behaviors, such as aggressive behavior, substance abuse, discipline behavior, and antisocial behavior [7,8].

Current research on parental control mostly focuses on immediate responses to parental control by children and adolescents. Few studies have involved emerging adults, which also shows the influence of parental control on their thinking and behaviors when emerging adults encounter different situations [9]. Adversarial growth describes the positive changes that individuals make after facing adversity [10], which may vary greatly due to parental styles and the family environment. In China, parents have a relatively high degree of control over their children [11]. Confucianism in China values the authoritarian role of parents and the self-repression of children. Especially after the 1980s, China began to implement a Family Plan Policy in order to curb the rapid growth of the population. Most families have only one child, and parents have high expectations of their children. As a result, many families more frequently show intrusive parenting styles, over-protection, and high levels of control over children [12,13] .

Do children who grow up in this controlling parenting style have a weaker self-identity, and are they able to maintain a positive and optimistic attitude in the face of adversity in adulthood, and benefit from that adversity? In particular, can individuals with a growth mindset inhibit the negative effect of parental control on young people’s individual self-perceptions? It will help parents to use control appropriately and better promote their children’s well-being through exploring the influence mechanism of parental control on individual adversarial growth and the moderating effect of a growth mindset.

## 2. Theoretical Background

Parental control is an important dimension of parenting style [14], in which parents impose their own thoughts and emotions on children in order to limit children’s psychological and behavioral autonomy [4]. Parents who force the children to obey the parents’ own thoughts by insisting on authority, triggering guilt, and withdrawing love will disturb the children’s inner world and cause psychological conflict [6]. Parental control can cause children to become overly dependent on parents, reduce their autonomy, and deprive children of opportunities to acquire problem-solving skills, so it is usually regarded by researchers as a negative parenting behavior [9]. Children desire to escape parental control under the development of their autonomy needs, but if parents limit children’s behavioral development by strengthening control, it will lead to poor parent–child relationships [15]. Some studies demonstrate that, when parents limit and supervise their children’s behavior by enforcing normative requirements, it is beneficial to individual development [16]. However, most empirical studies have shown that a high level of parental control will have a serious negative impact on the shaping and formation of a child’s personality, which is not conducive to the normal development of psychological functions [6]. A high level of parental control impairs children’s ability to be independent, hinders them from forming a safe and positive sense of self [17,18], and also makes them prone to aggressive behavior, antisocial behavior, drug abuse, internet addiction, and certain abnormal behaviors [19].

In the process of self-seeking development, the internal emotional state and the external social environment are integrated and coordinated with each other. Self-identity is formed in the process of thinking and making decisions about personal outlook on life, values, occupations, and ideals [20]. It is a clear and definite self-affirming cognition that an individual has in the process of exploring the path of life, including the goals and beliefs that the individual hopes to achieve. It reflects the individual’s inner purpose and sense of direction, which are crucial to the existence of the individual and enables individuals to obtain clear and stable goal values, as well as beliefs and self-affirmation [21]. The formation of self-identity needs to consider the influence of the social environment, which is a socialization process that emphasizes the individual’s survival in the social environment [20]. At the same time, self-identity is also significantly correlated with personal traits [22], such as extraversion and conscientiousness, and can significantly predict the level of individual self-identity [23,24]. Individuals with high self-identity can accept themselves and the outside world more easily, enjoy life more, and have lower anxiety [25]. They can better adapt to their environment and exhibit positive and social behaviors [26].

Some people experience physical and psychological harm as a result of adversity, such as anxiety, depression, and post-traumatic stress disorder [27]. However, there are also many people who have positive psychological changes after experiencing adversity [28]. When facing a life-threatening or challenging event, the individual reassesses their own goals and develops a new perspective on life that allows the individual to function at a higher level than before [10]. Improvements in relationships with others, the discovery of new possibilities, the enhancement of personal strength, and changes in attitudes towards life are all manifestations of adversarial growth and reflect positive psychological states [29,30]. Individual differences in coping with adversity are related to many factors, and an understanding of the influence mechanisms could allow individuals to consciously respond to adversity and improve mental health [31]. Higher social support can help individuals improve their confidence in dealing with difficult situations and promote positive change [32,33]. Different coping strategies in the face of adversity can also predict individual changes after adversity, and positive coping strategies are more likely to trigger individual adversarial growth [34,35]. Therefore, we propose Hypothesis 1.

**Hypothesis** **1.** *Parental control has a significant negative impact on adversarial growth.*

The family is the micro-system environmental factor that plays an important role in child development [36]. Parenting style is one of the most direct family factors [37]. Parental control impairs the development of adolescent autonomy and self-control [38], and easily leads to negative self-recognition and a decline in self-identity [39], which causes children to neglect their own emotions and suppress their thoughts, and hinders the acquisition of social skills [4,40]. Self-identity also affects an individual’s understanding of life meaning [41]. Individuals with high self-identity recognize their uniqueness when facing changes in socioeconomic status and are able to cope with life’s challenges by changing priorities and fully appreciating life at the present moment [42,43]. Therefore, higher parental control is not conducive to the development of the individual’s self-identity, thereby reducing the possibility of obtaining adversarial growth. Self-identity plays a mediating role in the relationship between parental control and adversarial growth. Therefore, we propose Hypothesis 2.

**Hypothesis** **2.** *Self-identity has a mediating role on the relationship between parental control and adversarial growth.*

Individuals with a growth mindset have a growth outlook, and they believe that their intelligence and abilities are unknown, growable, moldable, and regulated [44]. They are willing to change the status quo through personal efforts, and they believe that the challenges they encounter can help them learn and grow [45]. They regard great challenges as infinite possibilities for growth. Even if they fail in practice, they will not easily deny themselves, but solve problems in the process and continue to improve. Students with a growth mindset will work harder, be more engaged [46], and achieve better grades when faced with learning pressures [47]. At the same time, many empirical studies have also confirmed that the growth mindset is also significantly related to individual self-efficacy and career development [48,49], which can help individuals buffer the unfavorable external environment and improve self-concept [50,51]. Parenting styles can have different effects on an individual’s self-perception, and high-controlling parents can alter an individual’s self-evaluation and self-control [52]. Individuals with a growth mindset oppose thinking solidification, and believe that they can change the current situation through their own efforts [53,54], which can inhibit the negative impact of parental control on individual self-identity. They are also more likely to make positive efforts to grow when faced with adversity. Therefore, a growth mindset plays a moderating role on the mediating relationship between parental control, self-identity, and adversarial growth. Therefore, we propose Hypothesis 3 and 4.

**Hypothesis** **3.** *A growth mindset plays a negative moderating role on the relationship between parental control and self-identity.*

**Hypothesis** **4.** *A growth mindset plays a moderating role on the mediating relationship between parental control, self-identity, and adversarial growth.*

## 3. Method

### 3.1. Procedure and Participants

In China, university is the beginning of a child’s independence. Before that, children generally live with their parents and attend local schools. After high school, they can choose universities across the country and start living on campus. Therefore, on the one hand, the thinking and behaviors of college students have profound traces of their original family; on the other hand, they begin to face all the situations in life independently. Data were collected on parental control, self-identity, adversarial growth, and the growth mindset through questionnaires. All respondents were college students from Mainland China and the Hong Kong and Macao Special Administrative Regions.

In order to effectively reduce the common method bias, we selected two time periods to issue the questionnaires with a one-month interval. Time 1: T1 stage questionnaire data were collected through convenience sampling including parental control, the growth mindset, and control variables (gender, age, education, and major). A total of 500 questionnaires were distributed, 412 valid questionnaires were recovered, and the effective recovery rate of the questionnaire was 82.4%. Time 2: T2 stage questionnaire data were collected through matching numbers, including self-identity and adversarial growth. A total of 412 questionnaires were distributed, and 354 valid questionnaires were recovered. The effective recovery rate of the questionnaire was 85.9%. Respondents were clearly informed of the study purpose before answering the questionnaire and were assured that the data collected would only be used for academic research. Respondents could terminate the survey at any time. Incomplete questionnaires are considered invalid. The specific sample characteristics are shown in Table 1.

### 3.2. Measurement

The scales used in this study are all self-report scales. The variables were measured through a Likert 5-point scale from “completely disagree” to “completely agree.” Scores were averaged to create the composite scores for data analysis.

**Parental control** was measured with the control sub-dimension scale in the PBI (Parental Bonding Instrument) developed by Parker et al. [55], with a total of 5 items, such as “My parents try to control everything about me.” In this study, the Cronbach’s alpha coefficient of this scale was 0.967.

**Self-identity** was measured with the scale developed by OCHSE and PLUG [56], with a total of 19 items, such as “I know how to live.” In this study, the Cronbach’s alpha coefficient of this scale was 0.940.

**Adversarial growth** was measured with the scale developed by Cann et al. [57], with a total of 10 items, such as “I feel stronger than I imagined.” In this study, the Cronbach’s alpha coefficient of this scale was 0.971.

**The growth mindset** was measured with the scale developed by Dweck [44], with a total of 6 items, such as “I can change my intelligence level to a great extent.” In this study, the Cronbach’s alpha coefficient of this scale was 0.927.

**Control variables** included gender, age, education, and major, since individual perceptions of parenting styles and attitudes toward adversity vary in terms of these variables.

We used SPSS 26 and Mplus 8.3 for statistical analysis, which mainly involved reliability analysis, confirmatory factor analysis, correlation analysis, hierarchical regression, and structural equation analysis.

## 4. Results

### 4.1. Reliability and Validity

In this study, Mplus 8.3 software was used to conduct confirmatory factor analysis on the four main variables of parental control, the growth mindset, self-identity, and adversarial growth. The CFA results in Table 2 show that, compared with the single-factor model, the two-factor model, and the three-factor model, the overall fit of the four-factor model is better ($\chi^{2}/df$ = 3.036, RMSEA = 0.076, CFI = 0.952, TLI = 0.946, SRMR = 0.032). Therefore, there is good discriminant validity among the main variables of this study. The model fit of the single-factor model is far from acceptable ($\chi^{2}/df$ = 31.305, RMSEA = 0.293, CFI = 0.269, TL I = 0.196, SRMR = 0.370). Therefore, the common method deviation of the data in this study is not serious. The CR values corresponding to the research variables in the component validity test range from 0.918 to 0.970, which are all greater than 0.8 and indicate that the variables have good internal consistency. In terms of convergent validity, the AVE values of the study variables range from 0.693 to 0.866, which all meet the criterion of greater than 0.5.

### 4.2. Descriptive Statistical Analysis

The descriptive statistics and correlation coefficients of the variables in this study are shown in Table 3. Parental control and self-identity are significantly negatively correlated (*r* = −0.182, *p* < 0.01); parental control and adversarial growth are significantly negatively correlated (*r* = −0.191, *p* < 0.01); self-identity and adversarial growth are significantly positively correlated (*r* = 0.599, *p* < 0.01). The verification results initially supported the relationship of the research model.

### 4.3. Hypothesis Testing

As shown in Table 4, hierarchical regression was used to test the relationship between parental control, self-identity, adversarial growth, and the growth mindset. First, after controlling gender, age, education, and major, parental control had a significant negative influence on adversarial growth (β = −0.187, *p* < 0.001). Therefore, Hypothesis 1 is supported. Second, the mediating role of self-identity between parental control and adversarial growth was examined. After controlling gender, age, education, and major, parental control had a significant relationship with adversarial growth (β = −0.187, *p* < 0.001) and self-identity (β = −0.166, *p* < 0.01). When self-identity entered the regression equation as a mediating variable, the role of the mediating variable was still significant (β = 0.572, *p* < 0.001). Although the relationship between parental control and adversarial growth was still significant, the effect became weaker (β = −0.093, *p* < 0.05), so self-identity has a partial mediating effect between parental control and adversarial growth. Therefore, Hypothesis 2 is supported. Finally, after centralizing the related variables, parental control, the growth mindset, and their interaction were brought into the regression equation with self-identity as the dependent variable. Parental control (β = −0.228, *p* < 0.001), the growth mindset (β = −0.105, *p* < 0.05), and their interaction (β = −0.273, *p* < 0.001) had a significant influence, so a growth mindset has a negative moderating effect between parental control and self-identity. Specifically, a growth mindset can inhibit the influence of parental control on self-identity. Therefore, Hypothesis 3 is supported.

As shown in Table 5, after 5000 random samplings by the percentile-based bootstrap, the 95% confidence interval of the direct effect of self-identity between parental control and adversarial growth (−0.088, SE = 0.045) is [−0.164, −0.015], and the interval does not contain zero, which means the direct effect is significant. The 95% confidence interval of the indirect effect of self-identity between parental control and adversarial growth (−0.105, SE = 0.034) is [−0.162, −0.050], and the interval does not contain zero, which means the indirect effect is significant. Therefore, self-identity plays a partial mediating role on the impact of parental control on adversarial growth. Parental control negatively affects an individual’s adversarial growth through self-identity.

Finally, 5000 bootstrap samples were used to produce an unbiased estimate of the effect of parental control on adversarial growth through self-identity with a weak growth mindset (Mean-1SD) and a strong growth mindset (Mean + 1SD) (Table 6). With a weak growth mindset, the 95% confidence interval (−0.071, SE = 0.038) is [−0.148, 0.000], and the interval does contain zero. The mediating effect does not exist with a weak growth mindset. With a strong growth mindset, the 95% confidence interval (−0.256, SE = 0.053) is [−0.361, −0.150], and the interval does not contain zero. The mediating effect does exist with a strong growth mindset. The 95% confidence interval of the moderated mediating effect (−0.121, SE = 0.030) is [−0.176, −0.059], and the interval does not contain zero. The moderated mediating effect does exist. A strong growth mindset weakens the influence of parental control on adversarial growth through self-identity. Therefore, Hypothesis 4 is supported.

## 5. Conclusions and Discussion

The parenting style will affect the individual’s self-cognition and behavioral choices, especially for college students who are gradually entering adulthood. The influence of the original family is still deeply imprinted on them [58]. This study conducted a questionnaire survey on 354 college students born in one-child families in China to explore the impact mechanism of parental control on adversarial growth and verify the moderating role of a growth mindset. Statistical analysis shows that parental control negatively affects adversarial growth, and self-identity plays a mediating role between parental control and adversarial growth. A higher degree of parental control will reduce an individual’s sense of self-identity, which is not conducive to the occurrence of adversarial growth. A growth mindset negatively moderates the relationship between parental control and self-identity, and a growth mindset negatively moderates the indirect effect of parental control on adversarial growth through self-identity. Individuals with a growth mindset may reduce the negative effects of parental control on self-identity and adversarial growth.

For college students, as emerging adults, although most of them have been independent from their original families, this study confirms in an Asian sample that parental control can still affect their perceptions and behaviors. Especially in adversity, differences in parenting styles lead to different individual responses. College students who grew up in families with a high level of parental control tend to have lower self-identity and find it easy to withdraw when faced with difficulties [39]. They want to seek dependence, especially help from their parents, so they usually lack the efforts to actively cope and change, which reduces the possibility of growth from adversity. This study also verifies the important role of a growth mindset, as have many previous studies. Individuals with a growth mindset appreciate the value of effort more and believe that they can change the current situation through their own efforts. A growth mindset attenuates the negative effects of parental control to a certain extent.

The role of parental controls has been debated in academia for a long time. Some studies have verified the positive effect of parental control in collectivist cultures, since it can provide children with improved restraints and guidance to help them socialize [16]. However, from a developmental perspective, the negative effects of parental control are more pronounced [59]. Firstly, all parents have high expectations of their children and want to give them adequate guidance and support through different parenting behaviors [60], but they need to use controls carefully. In particular, countries with a high level of collectivism tend to value family culture more, which emphasizes children’s obedience to their parents, and parents limit and supervise children’s behaviors and guide children to avoid negative behaviors [61]. Children’s independent thinking and personality development may be inhibited due to strict parental control. While accepting parents’ restrictions on their behavior, children are also reflecting on the correctness of their own behaviors. When parents’ requirements are inconsistent with their own thoughts, parental control is actually a denial of children, which will negatively affect their self-identity and reduce their confidence in dealing with problems independently [62]. When they face difficulties, a high level of dependence can inhibit their initiative, so children will be less able to take initiative in facing adversity. It is suggested that parents should consider providing their children relative autonomy. In the case of ensuring that the risks are manageable, parents should help their children rethink and actively change after experiencing setbacks, rather than strictly controlling them in advance.

Secondly, parents should consciously help their children learn to be independent even in countries with a collectivism culture. College is a transitional stage for teenagers to become mature. They begin to try to live independently, face difficulties in learning, and establish their own interpersonal network [63]. At this time, instead of letting the child report everything from time to time, parents should give their children full trust, avoid too much remote control, and provide support when the child needs help, which is more conducive to the growth of the child. In this age of increasing emphasis on individuality and innovation, individuals accustomed to obedience may not be able to be autonomous and perform challenging tasks [64]. Teaching such independence is a gift that parents can give their children to help them face the world on their own.

Finally, parents should actively guide their children to develop a growth mindset and transmit the idea that, through hard work and learning, the status quo can be changed. The growth mindset is a positive personal trait that reflects an individual’s confidence in self-control and the environment [45]. Even if the individual with a growth mindset level encounters adversity or trauma in the future, he can still proactively respond to crises and suppress negative effects. The growth mindset can help individuals learn new skills and gain new development amid adversity. It is a valuable asset that children can benefit from for their entire lives.

There are certain limitations to this research. First, many scholars discuss parental control from two dimensions, psychological control and behavioral control, and believe that it will have different effects on individuals’ self-perception and behaviors. However, whether there is a clear boundary between the two parental controls is still academically controversial [65]. Therefore, no distinction was made in discussion between psychological control and behavioral control in this study, and there may be some bias. The sample of this study is only 354. Although it basically meets the sample size requirements of confirmatory factor analysis, regression analysis, and structural equations, it is still a small sample survey, which may affect the overall validity of the study. Finally, the respondents in the study are all from one-child families in China. Chinese traditional culture advocates authority, and family culture emphasizes children’s obedience to parents [66]. The implementation of the one-child policy has increased the expectations of Chinese parents on their children [12], so Chinese parents use intrusive parenting styles more frequently than western parents [11]. The research conclusions are based on a Chinese sample, which are quite different from countries with an individualistic culture. Therefore, the conclusions of this study may have certain external validity problems.

A growth mindset in the face of adversity is a valuable asset in life, and it is also a complex social phenomenon. Its influencing factors and formation mechanisms are worthy of further exploration. Family environments, parenting styles, peer relationships, organizational support, community environment, and personality traits may all predict an individual’s growth when coping with adversity. In future research, we will continue to explore the influence of the family and parenting styles on individual growth in different cultures, and conduct cross-cultural comparative research.

## Figures and Tables

**Table 1:** Sample characteristics (*N* = 354).

Characteristics	Percent	Number
**Gender**		
Male	57.9%	205
Female	42.1%	149
**Age**		
20 years old or below	52.5%	186
21 to 25 years old	40.7%	144
26 to 30 years old	4.8%	17
31 years old or above	2.0%	7
**Education**		
Undergraduate students	85.0%	301
Graduate students	15.0%	53
**Major**		
Humanities	51.9%	184
Science and technology	48.1%	170

**Table 2:** Confirmatory factor analysis (*N* = 354).

Model	Factors	$\chi^{2}/\mathrm{df}$	RMSEA	CFI	TLI	SRMR
Four-factor model	PC, SI, GM, AG	3.036	0.076	0.952	0.946	0.032
Three-factor model	PC + SI, GM, AG	10.387	0.163	0.777	0.751	0.231
Two-factor model	PC + SI + GM, AG	13.853	0.191	0.691	0.659	0.244
Single-factor model	PC + SI + GM + AG	31.305	0.293	0.269	0.196	0.370

Note: PC: Parental Control; SI: Self-Identity; AG: Adversarial Growth; GM: Growth Mindset.

**Table 3:** Mean, standard deviation and correlation statistics (*N* = 354).

	Mean	SD	1	2	3	4	5	6	7	8
Gender	1.421	0.494								
Age	1.562	0.680	0.297 **							
Edu	1.150	0.357	0.155 **	0.609 **						
Major	1.554	0.629	0.305 **	0.536 **	0.122 *					
PC	2.328	0.863	−0.042	0.018	0.129 *	−0.096	**(0.931)**			
SI	3.429	0.665	0.028	0.171 **	0.060	0.279 **	−0.182 **	**(0.832)**		
AG	3.560	0.597	0.008	0.101	0.075	0.196 **	−0.191 **	0.599 **	**(0.858)**	
GM	3.033	0.769	−0.120 *	−0.170 **	−0.095	−0.185 **	−0.202 **	−0.055	−0.012	**(0.927)**

Note: ** *p* < 0.01, * *p* < 0.05; PC: Parental Control, SI: Self-Identity; AG: Adversarial Growth; GM: Growth Mindset. The bold value on the diagonal is the AVE root value of each variable.

**Table 4:** Hierarchical regression (*N* = 354).

	AG			SI			
	M1	M2	M3	M4	M5	M6	M7
Gender	−0.060	−0.066	−0.020	−0.024	−0.069	−0.074	−0.075
Age	−0.069	−0.071	−0.085	−0.086	0.028	0.025	0.046
Education	0.097	0.126	0.086	0.100	0.020	0.045	0.003
Major	0.240 ***	0.222 **	0.074	0.069	0.283 ***	0.267 ***	0.248 ***
PC		−0.187 ***		−0.093 *		−0.166 **	−0.228 ***
SI			0.588 ***	0.572 ***			
GM							−0.105 *
PC*GM							−0.273 ***
ΔR^2^	0.047	0.034	0.317	0.325	0.083	0.026	0.070
F	4.308 ***	6.141 ***	39.876 ***	34.302 ***	7.872 ***	8.551 ***	10.779 ***

Note: *** *p* < 0.001, ** *p* < 0.01; * *p* < 0.05; PC: Parental Control, SI: Self-Identity; AG: Adversarial Growth; GM: Growth Mindset.

**Table 5:** Percentile-based bootstrap random sampling results of 5000 times (*N* = 354).

	Estimate	S.E.	Est./S.E.	LLCI	ULCI
Total Effect	−0.193	0.060	−3.200	−0.292	−0.090
Direct Effect	−0.088	0.045	−1.932	−0.164	−0.015
Indirect Effect	−0.105	0.034	−3.065	−0.162	−0.050

**Table 6:** Moderated mediation effect (*N* = 354).

	Mediating Effect: Parental Control → Self-Identity → Adversarial Growth					
Moderator	Coefficient				95% CI	
Growth Mindset	Effect	S.E.	Est./S.E.	*p*	LLCI	ULCI
Weak	−0.071	0.038	−1.844	0.065	−0.148	0.000
Strong	−0.256	0.053	−4.804	0.000	−0.361	−0.150
IMM	−0.121	0.030	−4.088	0.000	−0.176	−0.059

Note: IMM: Index of Moderated Mediation, CI: Confidence Interval.

## Data Availability

The data presented in this study are available from the corresponding author on request.
